# A milk formula containing maltodextrin, *vs*. lactose, as main carbohydrate source, improves cognitive performance of piglets in a spatial task

**DOI:** 10.1038/s41598-018-27796-1

**Published:** 2018-06-21

**Authors:** Caroline Clouard, Cindy Le Bourgot, Frédérique Respondek, J. Elizabeth Bolhuis, Walter J. J. Gerrits

**Affiliations:** 1 Wageningen University & Research, Department of Animal Sciences, Adaptation Physiology Group, P.O. Box 338, 6700 AH Wageningen, The Netherlands; 2 Tereos S.A., R&D Department, Z.I. et portuaire B.P. 32, 67390 Marckolsheim, France; 3 Wageningen University & Research, Department of Animal Sciences, Animal Nutrition Group, P.O. Box 338, 6700 AH Wageningen, The Netherlands

## Abstract

In recent years, lactose-free and low-lactose infant formulas have been increasingly used. The impact of using different carbohydrates than lactose on later cognition of formula-fed infants remains, however, unknown. We examined the effects of providing formulas containing either digestible maltodextrin or lactose as main carbohydrate source (28% of total nutrient composition) on cognitive performance of piglets. Piglets received the formulas from 1 to 9 weeks of age and, starting at 12 weeks, were individually tested in a spatial holeboard task (*n* = 8 pens/formula), in which they had to learn and memorize a configuration of baited buckets. After 28 acquisition trials, piglets were subjected to 16 reversal trials in which the location of the baited buckets was changed. Piglets fed the maltodextrin-based formula had higher reference memory (RM) scores than piglets fed the lactose-based formula towards the end of acquisition. During the switch of configuration, piglets offered the maltodextrin-based formula tended to have higher RM scores and make fewer RM errors than piglets offered the lactose-based formula. Working (short-term) memory was not affected by the formulas. Compared to lactose, the use of maltodextrin in milk formulas improved long-term spatial memory of piglets, even weeks after the end of the intervention.

## Introduction

Lactose is the primary carbohydrate in human breast milk and milk-based formulas for infants^1^. The specific need for lactose, however, has not been proven^2^ and, in recent years, lactose-free or reduced-lactose formulas have been increasingly used^3^. This increase in the use of infant formulas containing alternative carbohydrate sources is mainly driven by the increasing concern surrounding lactose intolerance in infants^4^. In the European Union, some authorized glycaemic carbohydrates, like pre-cooked starch, gelatinized starch, and maltodextrin, are commonly used in infant and follow-on formulas as alternative sources of carbohydrates^5^. Digestible maltodextrin is a low-sweet saccharide polymer consisting of D-glucose units primarily linked linearly with α-1,4 bonds; it also has a branched structure through α-1,6 bonds. Similar to lactose, maltodextrin has an approximate energy value of 4 kcal/g, and is the main carbohydrate source in non-allergenic infant formulas containing non-dairy ingredients^6^. The use of maltodextrin as a source of digestible carbohydrates in infant formulas has been suggested to help reduce osmotic load and related intestinal distress^6^, while having no adverse effects on growth^7^. Although maltodextrin is thus generally considered safe for infant development, little is known on the long-term consequences of replacing lactose with maltodextrin for health and biological functioning later in life.

Early nutrition can influence development and may result in long-lasting adaptations of metabolic, immune, behavioural and brain functions in later life, a phenomenon known as nutritional programming^8–12^. Carbohydrates, notably, are one of the primary sources of energy for the development and growth of infants, and glucose, obtained from the digestion and absorption of complex carbohydrates, is a major energy source for perinatal brain development under normal physiological conditions in humans and animals^13,14^. The quality and quantity of carbohydrates consumed in early life have been found to shape offspring’s metabolic profile^15^, but also food preferences and the consumption of sweet, high-energy food later in life^16^. Surprisingly, however, little is known on the relation between early carbohydrate intake and neuro-cognitive development^16^. Yet, increased carbohydrate intake in the immediate postnatal life results in behavioural, metabolic, and physical alterations in rats (*e.g*. hyperphagia, hyperinsulinemia and weight gain)^12^. Increasing the intake of carbohydrates with a low glycaemic index during pregnancy also improves maternal glucose homeostasis, as well as insulin and glucose regulation in the offspring^15^. Furthermore, infants fed glucose syrup-containing formulas show different postprandial metabolic responses than infants fed lactose-containing formulas, with, notably, alterations of blood glucose levels^17^. If carbohydrate sources ingested during a sensitive period of (brain) development influence (long-term) metabolic functioning of the infant, it may also have long-lasting consequences on brain development and cognitive function. To the best of our knowledge, however, no studies have yet investigated the impact of replacing lactose with another source of carbohydrates on later life cognitive functions in formula-fed infants.

This project aimed to examine the effects of formulas containing maltodextrin or lactose as the main source of carbohydrates on later life cognitive performance of piglets. Growth and preferences of pigs for sweet caloric or non-caloric solutions were also assessed to evaluate the potential impact of motivational factors on performance during the holeboard task. The pig, an omnivorous species with high cognitive abilities^18–20^, shares multiple similarities with humans in terms of nutritional needs, as well as brain development, physiology and function^21–23^, and is therefore an excellent animal model to study the impact of early nutrition on cognitive development of human infants^23,24^.

## Methods

**Figure 1:**
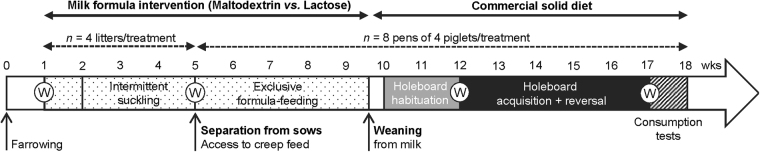
Study timeline. W stands for weighing.

This experiment was performed in accordance with the Dutch law on animal experimentation, which complies with the European Directive 2010/63/EU on the protection of animals used for scientific purposes. The Animal Welfare Body of Wageningen University & Research has approved this research. The study timeline is shown in Fig. 1.

### Animals and housing

Eight multiparous sows (Topigs 20) and their litters (Tempo × Topigs 20) from the Swine Innovation Centre of Wageningen University & Research (VIC, Sterksel, The Netherlands) were used. Lactating sows and their litters were housed in individual farrowing pens (240 × 180 cm) equipped with a farrowing crate. A jute bag was provided around parturition to be used as nesting material. Rooms had a natural light-dark cycle. Ambient temperature at arrival of the sows to the farrowing unit, one week before the expected farrowing date, was 25 °C and was decreased over lactation *via* a floor cooling system. From birth onwards, piglets had access to a heated area. Between 24 and 48 hours after birth, if needed, piglets were cross-fostered between sows to balance litter sizes based on the number of functional teats (12.7 ± 0.26 piglets/litter after cross-fostering).

Sows and their litters were kept together continuously in the farrowing unit until 2 weeks after the expected farrowing date (15 ± 0.5 days after birth). From 2 to 5 weeks of age, the piglets were subjected to an intermittent suckling (IS) regimen, during which sows were separated from their litter for 8 h/day (7:30 to 15:30). During separation, sows were housed individually in a separated room (IS unit) to prevent visual and auditory contact with their piglets. This procedure allowed to start the dietary intervention at a very young age, thus stimulating the intake of the formulas, while avoiding stress-related developmental issues that can be caused by a total and early separation from the sow. During lactation, sows were fed twice daily (1 meal in the morning in the IS unit and 1 meal in the afternoon after return to the farrowing unit) a standard commercial diet according to the normal net nutrient recommendations for lactating sows.

At about 5 weeks of age (36 ± 0.5 days after birth), piglets were separated from the sows. At separation, 8 healthy piglets (4 females and 4 males) with birth weights closest to the average litter birth weight were selected from each of the 8 litters and were transported to the experimental farm of Carus (Wageningen University & Research, The Netherlands). The 64 selected piglets were housed in groups of 4 littermates (no mixing), with 2 females and 2 males per pen (280 × 180 cm), in 2 identical and adjacent rooms. Pens were equipped with a chain with screws attached to it as enrichment material. Fresh wood shavings were added daily to the pens as bedding. Fresh straw was added daily to the pens from the end of the dietary intervention onwards, at about 9–10 weeks of age (67 ± 0.5 days after birth). Room temperature at arrival of the piglets was 25 °C and was then gradually decreased and kept at 21 °C until the end of the experiment. Lights were on and a radio was playing in the background from 7:00 to 19:00 daily.

### Formulas and diets

Two milk formulas differing in their main carbohydrate source were formulated. The lactose-based formula was designed to have a macronutrient composition resembling that of the milk of sows in mid-lactation^25^, but with slightly lower levels of proteins and fat to allow inclusion of 28% lactose as the main carbohydrate. The maltodextrin-based formula consisted in the same formula in which lactose was replaced with maltodextrin (28% of nutrient composition; Dextrose Equivalent (DE) 19). The formulas were prepared by mixing milk powder in lukewarm water (~45 °C), with a milk powder to water ratio of 1:4. Ingredient and nutrient composition of the milk powders are presented in Table 1.

**Table 1:** Ingredient and nutrient compositions of the experimental milk powders.

	Lactose	Maltodextrin
Ingredient composition (%)		
Basal powder mix^a^	75.0	75.0
Lactose	25.0	—
Maltodextrin	—	25.0
Calculated nutrient composition (per 100 g)		
ME (MJ)	2.15	2.15
NE (MJ)	1.73	1.73
Protein (g)	25.0	25.0
Fat (g)	32.0	32.0
Carbohydrates (g)	31.0	32.3
of which lactose	28.0	4.30
of which maltodextrin	3.00	28.0
Crude ash (g)	6.80	6.80

^a^Composition (% relative to total ingredient composition of the experimental milk powders): fat concentrate I (80% coconut oil on maltodextrin), 13%; fat concentrate II (80% palm oil on sweet whey), 24.65%; WPC-75%CP, 25%; wheat protein hydrolysed, 5.02%; Di-potassium phosphate 17.7%P, 1.5%; citric acid, 1%; sodium chloride, 1%; calcium carbonate, 0.75%; premix, 0.50%; calcium formate, 0.50%; calcium acetate, 0.50%; magnesium sulphate, 0.50%; potassium sorbate, 0.34%; L-lysine HCL 98%, 0.30%; DL-methionine 99%, 0.20%; silica, 0.06%; L-tryptophan 98%, 0.05%; sweetener, 0.03%; flavour, 0.03%; vitamin E, 0.02%; iron sulphate, 0.02%; L-threonine 98%, 0.01%; copper sulphate, 0.01%.

Piglets had access to the sow milk only for the first week of life. At 1 week of age, piglets were allocated to 1 of the 2 milk formulas and received the formulas until 9–10 weeks of age (*i.e*. from 8 to 67 days after birth), resulting in *n* = 4 litters per treatment before separation from the sow (1 to 5 weeks of age), and *n* = 8 pens of 2 male and 2 female piglets per treatment after separation (5 to 9–10 weeks of age). Allocation of the litters to the formulas was balanced for sow parity, average litter size, and weight at birth. Treatments were distributed in a randomized design within rooms before separation from the sows, and within and between rooms after separation from the sows. Before separation from the sow (at 5 weeks of age), and during the week directly following separation from the sow, piglets were allowed *ad libitum* access to the formulas. Between 7 and 9 weeks of age, piglets were housed for 72 hours in climatized respiration chambers for the continuous measurement of O_2_ consumption and production of CO_2_, from which heat production, activity-related heat production and thermic effect of feeding were calculated (article in preparation). To this end, formulas were provided restrictedly at 2.5 × the net energy requirements for maintenance^26^ between 6 and 9 weeks of age. Throughout the period of restricted feeding, formulas were distributed in two meals, at approximately 8:30 and 16:30. Piglets were fed the formulas *via* milk dispensers, equipped with a single 5-L tank connected to 1 drinking cup (200 mL) before separation from the sows (1 to 5 weeks of age), and to 4 drinking cups mounted in series after separation from the sows (5 to 9 weeks of age). The drinking cups were equipped with a nipple allowing the milk to flow into the cup when touched by the piglet, while preventing overflow. Milk dispensers, pipes, and cups were cleaned daily with hot water, weekly with a basic solution, and monthly with an acidic solution.

After separation from the sow, at 5 weeks of age, piglets also had restricted access to a commercial creep feed (250 g/pen/day) for 2 h/day to habituate them to solid feed. At 67 days of age, piglets were weaned from the milk formula to a commercial starter diet (172 g of crude protein per kg, Agruniek Rijnvallei, Wageningen, The Netherlands), over a 3-day transition phase. The starter diet was then offered to the piglets *ad libitum* until the end of the experiment. Piglets had *ad libitum* access to water throughout the whole experiment.

### Measurements

To assess the effect of formulas on piglet growth, piglets were weighed individually at birth, at the start of the dietary intervention, at separation from the sows, and at the start and end of holeboard testing, *i.e*. at 1, 5, 12 and 17 weeks of age, respectively (Fig. 1).

#### Spatial cognitive holeboard task

From 12 to 17 weeks of age, 2 piglets (1 male and 1 female) per pen (*n* = 8 pens/treatment) were individually tested in a spatial holeboard task, to assess learning, working and reference memory, as well as cognitive flexibility, that is the ability to respond to a new spatial configuration, also called ‘reversal’^27^.

**Figure 2:**
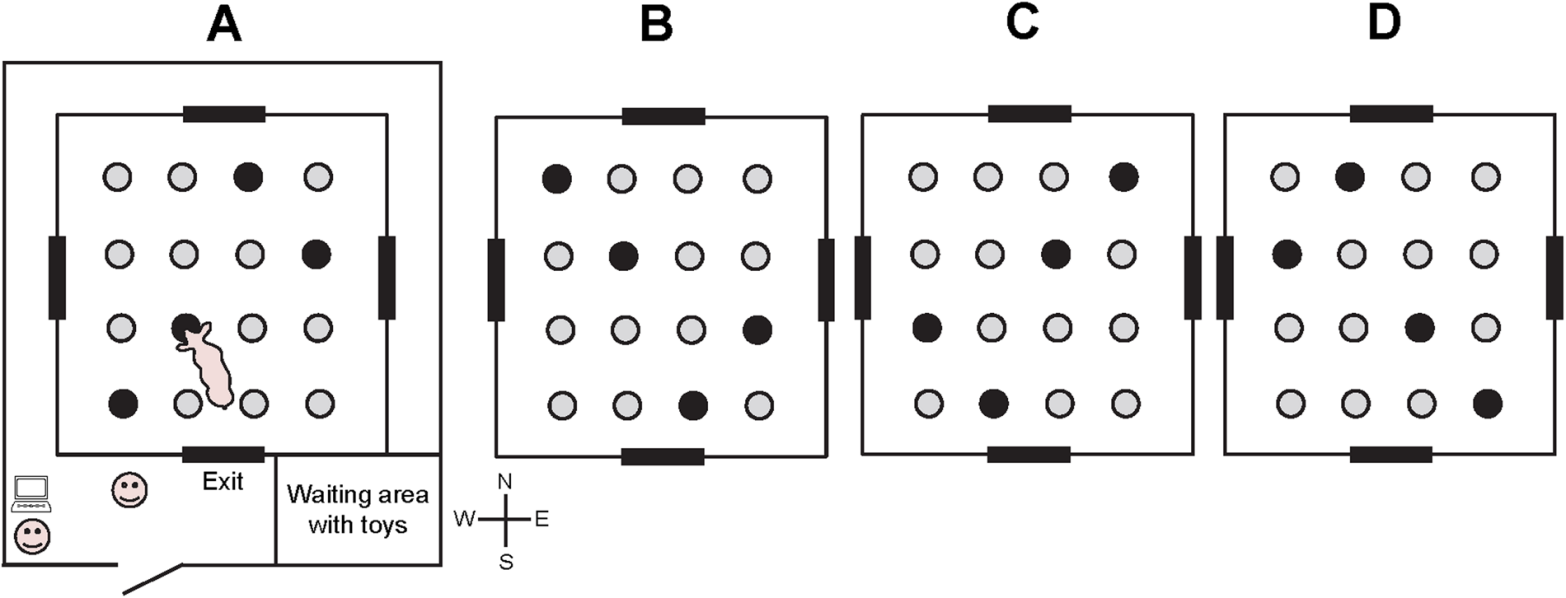
Schematic of the testing room and the four configurations of baited buckets for the holeboard task. Piglets were assigned to a fixed configuration of baited buckets during the acquisition phase, and to another configuration during the reversal phase (**A** to **C**,**B** to **D**,**C** to **A** and **D** to **B**).

The test arena (5.3 × 5.3 m) had black, wooden, 80-cm-high walls and 4 entrances with guillotine doors. The arena was surrounded by a corridor, and a waiting area (in the south-east corner of the room) containing a jute bag and some toys that were changed daily. In the arena, 16 grey metallic buckets (diameter: 19 cm) were screwed to the floor in a 4 × 4 matrix (Fig. 2). During the test, 4 of the buckets were baited with a chocolate raisin. To prevent the use of visual cues to find the rewards, the chocolate raisins were hidden under a thin layer of ground straw. All buckets also had a perforated false bottom under which fresh chocolate raisins were placed at the start of the day to prevent the use of odour cues to locate the baited buckets. Two experimenters (scoring the behaviours and controlling the guillotine doors) were standing on the south-west corner of the room and were visible to the test pig from the holeboard arena, thus constituting a visual cue for navigation (Fig. 2). Additional visual cues for navigation were a wooden door on the north wall of the room, and a yellow water hose hanging on the wall, close to the door. Piglets were deprived from feed overnight during the 3 phases of holeboard testing (habituation, acquisition and reversal).

From 10 to 12 weeks of age (Fig. 1), all piglets were gradually habituated to the experimenters, the buckets and rewards, the corridor leading to the test room, the testing room and the task in sessions of 10–15 min per day for 13 working days (*i.e*. excluding week-ends): after 2 days of habituation to the experimenters, buckets and food rewards in the home pens, the 4 piglets of each pen were allowed to explore the holeboard arena, in which the 16 buckets were baited, with their pen mates for 10–15 min per day for 4 days. After this phase, only 2 piglets per pen were subjected to individual habituation trials. The 2 piglets that were to be tested in the holeboard were selected just after separation from the sow as the 2 piglets (1 male, 1 female) with birth weights closest to the average pen birth weight. If a selected test piglet, however, systematically rejected the chocolate raisins, showed extreme stress responses (high-pitch vocalizations, standing alert, escape attempts) or did not perform the task at all (*i.e*. not looking in the buckets) at the end of the 4 first days of habituation, it was excluded from the selection, and replaced by the other pen mate of the same sex. The selected piglets were then allowed to individually explore the holeboard arena: for 3 days, the 16 buckets were baited, and for 4 days, only 8 buckets were baited. During the individual habituation trials, piglets were subjected to at least 2 trials (maximum 180 s per trial) per day.

After the habituation phase, the acquisition phase started (Fig. 1). Piglets were individually subjected to 2 massed trials (*i.e*. performed a few minutes apart) per day on 14 consecutive working days, *i.e*. 28 acquisition trials. While a piglet was tested, its pen mates were kept in the waiting area to minimise the stress induced by social isolation. The trial started when the piglet had its 4 legs in the testing arena and ended when the piglet found all 4 rewards or after 180 s. Every time the piglet visited a baited bucket for the first time, a clicker sound was produced to facilitate learning. If the piglet completed the task (*i.e*. found the 4 rewards in less than 180 s), a bike bell was rang, the exit guillotine door was opened, and the piglet was congratulated (“good job!”, “well done!”), and received a piece of apple. If the piglet did not complete the task within 180 s, a police siren sound was produced (distinctively different from the bike bell sound); the piglet was not congratulated and did not receive a piece of apple. After each trial, the piglet was led back into the waiting area with its pen mates. After 2 piglets per pen had been tested twice, all the pen mates were led back into their home pen and their feeder was opened. After each trial, faeces were removed and urine was wiped from the testing arena. In total 4 different configurations of baited buckets were used (Fig. 2). Each piglet was tested on a fixed configuration of baited buckets throughout the acquisition phase, with the configuration of baited buckets differing between the 2 piglets within each pen, and being balanced between formulas and rooms. Testing order within and between pens was alternated within and between days to balance for formulas. Different entrances were used daily, with two different entrances per day of test (*i.e*. 1 entrance per trial), to prevent piglet from developing a fixed pattern of visits that would reduce the working memory (WM) load^20^.

After the acquisition phase was completed, piglets were tested in a reversal phase, with 16 reversal trials in 8 working days. The procedure was the same procedure as that of the acquisition phase, but piglets were assigned to a different configuration of baited buckets (Fig. 2).

The following parameters were scored live using The Observer XT (Noldus Information Technology, Wageningen, The Netherlands): all visits and revisits to all buckets, latencies to all bucket visits, trial duration, total number of defecations, urinations and escape attempts during the trial. From the parameters recorded during the test, the following variables were calculated *a posteriori* according to van der Staay *et al*.^27^: working memory (WM) score (*i.e*., short-term memory) is the ratio between the number of rewarded visits and the number of visits and revisits to the baited set of buckets; reference memory (RM) score (*i.e*., long-term memory) is the ratio between the number of visits and revisits to the set of baited buckets and the number of visits and revisits to all buckets; WM errors is the number of revisits to baited buckets; RM errors is the number of visits and revisits to unbaited buckets; trial duration is the time needed to complete the task, *i.e*. latency to fourth baited bucket or 180 s; inter-visit interval (IVI) is calculated as (time to last bucket visit − latency to first bucket visit)/(number of bucket visits − 1); total number of visits and revisits.

#### Single-solution consumption tests

Between 17 and 18 weeks of age, piglets were subjected to 2 consecutive single-solution consumption tests to assess whether the main source of carbohydrates in early life affects attractiveness for caloric and non-caloric sweet solutions later in life. On 2 consecutive days, piglets of half of the pens were given access to a 10% (w/w) sucrose solution, and piglets of the other half of the pens to a 0.125% (w/w) sucralose solution for 30 min/day (10:00–10:30). After the first 2-day test, piglets were offered access to the other solution for another 2-day test; the 2-day tests were separated by the week-end. Order of solution distribution was balanced for treatment and room. On each day of testing, the piglets were deprived from feed for 2.5 hours before starting the test. The solutions were distributed in the milk dispensers used previously. In each pen, piglets were allowed free access to the starter feed as soon as the dispenser was filled with the solution. If needed, the dispensers were refilled during the tests so that dispensers were never empty during the 30-min tests. Solution refusals were weighed at the end of the 30-min tests and the dispensers were cleaned with hot water. Starter feed consumption was measured 4 hours after the feeders were opened (14:00). Solution and feed intake per pen were expressed in kg per kg of body weight.

### Statistical analysis

Data were analysed with SAS 9.1.3 (Statistical Analysis Software; SAS Institute, Cary, NC, USA). Model residuals were checked for normal distribution using the Shapiro-Wilk test. If model residuals were not normally distributed, data were transformed for analyses. Data are presented as (untransformed) means ± SEM.

Birth weight was analysed using a MIXED procedure with formula (maltodextrin, lactose) as fixed effect and pen nested within formula as random effect. Litter size at birth was included as a covariate. Weights after separation from the sow were analysed using a repeated MIXED procedure with weight over weeks taken as repeated measures. Formula (maltodextrin, lactose), week (5, 12 and 17 weeks of age) and their interaction were included as fixed effects and pen nested within formula as random effect. Birth weight was included as a covariate.

Growth (*i.e*. average daily weight gain, g/d) before and after separation from the sow was analysed using a MIXED procedure with formula (maltodextrin, lactose) as fixed effect and pen nested within formula as random effect. Litter size at birth, included as a covariate in the initial model for ADG before separation from the sow, had no effect on any variables and was removed from the final model. Weight at separation from the sow was included as a covariate in the model for ADG after separation from the sow.

In total, 7 test piglets were excluded from the analyses of the holeboard data because they showed extreme stress responses (1 maltodextrin piglet), refused to eat chocolate raisins even when offered by hand (1 pen of 2 maltodextrin piglets, 2 lactose piglets), had extreme diarrhoea at the start of the acquisition, thus not performing the task (1 lactose piglet), or died during the period of test (1 maltodextrin piglet). This resulted in *n* = 8 pens (13 piglets) for the lactose treatment group, and *n* = 7 pens (12 piglets) for the maltodextrin treatment group. Means of 4 consecutive trials were calculated, resulting in 7 and 4 trial blocks for acquisition and reversal phases, respectively. Data of the acquisition and reversal phases were analysed using a MIXED procedure with formula as fixed effect, time (*i.e*. block of trials) as linear effect, and pen nested within formula as random effect. Furthermore, difference in performance between the last block of acquisition trials (block 7) and the first block of reversal trials (block 8), *i.e*. transition phase, was assessed using a MIXED procedure with formula, block of trials and their interaction as fixed effect, and pen nested within formula as random effect. The difference in performance during this transition phase represents a measure of cognitive flexibility after the switch of configuration^28,29^. Sex, included as a fixed effect in the initial models, and its interaction with formula, had no effect on any of the variables and was removed from the final models.

In total, 2 piglets (1 maltodextrin piglet and 1 lactose piglet) died before the implementation of the single-solution consumption tests, resulting in *n* = 8 pens (31 piglets) for each treatment group. Data from the single-solution consumption tests (solution and feed intake) were expressed in kg per kg of body weight. Data were analysed using a MIXED procedure with formula (maltodextrin, lactose), solution (sucrose, sucralose), order of presentation (sucrose first, sucralose first) as fixed effects, weight at 17 weeks (before the start of the tests) as covariate, and pen nested with formula as random effect.

### Data availability

The datasets generated and/or analysed during this study are not publicly available due to patent filing but are available from the corresponding author on reasonable request.

## Results

### Growth

Average weight at birth did not differ between treatment groups (maltodextrin: 1.24 ± 0.11 kg, lactose: 1.35 ± 0.10 kg, *p* = 0.87). From separation from the sow to the end of holeboard testing, weight increased significantly over weeks (*p* < 0.001), but was not affected by formula × week interaction (5 weeks, maltodextrin: 9.86 ± 0.30 kg, lactose: 10.5 ± 0.30 kg; 12 weeks, maltodextrin: 31.6 ± 0.60 kg, lactose: 34.2 ± 1.11 kg; 17 weeks, maltodextrin: 58.1 ± 1.35 kg, lactose: 63.4 ± 1.87 kg, *p* = 0.93). Irrespective of the week, maltodextrin-fed piglets tented to be lighter than lactose-fed piglets (maltodextrin: 33.2 ± 0.74 kg, lactose: 36.04 ± 0.85 kg, *p* = 0.06). However, the formula treatment did not affect the average daily rate of body weight gain of piglets before or after separation from the sow (Table 2).

**Table 2:** Average daily gain (ADG, g/d) of piglets fed milk formulas containing lactose or maltodextrin as the only carbohydrate source from 1 to 9 weeks of age.

	Maltodextrin	Lactose	Effect of milk formula (*p*-values)
**Before separation from the sow** ^**a**^			
1 to 5 weeks of age	242 ± 10.0	264 ± 10.4	0.18
**After separation from the sow** ^**b**^			
5 to 12 weeks of age	444 ± 11.0	481 ± 14.0	0.40
12 to 17 weeks of age	791 ± 41.9	872 ± 32.1	0.34

^a^*n* = 4 litters per treatment.

^b^*n* = 8 pens (of 3 to 4 piglets) per treatment.

### Spatial cognitive holeboard task

#### Acquisition phase

**Figure 3:**
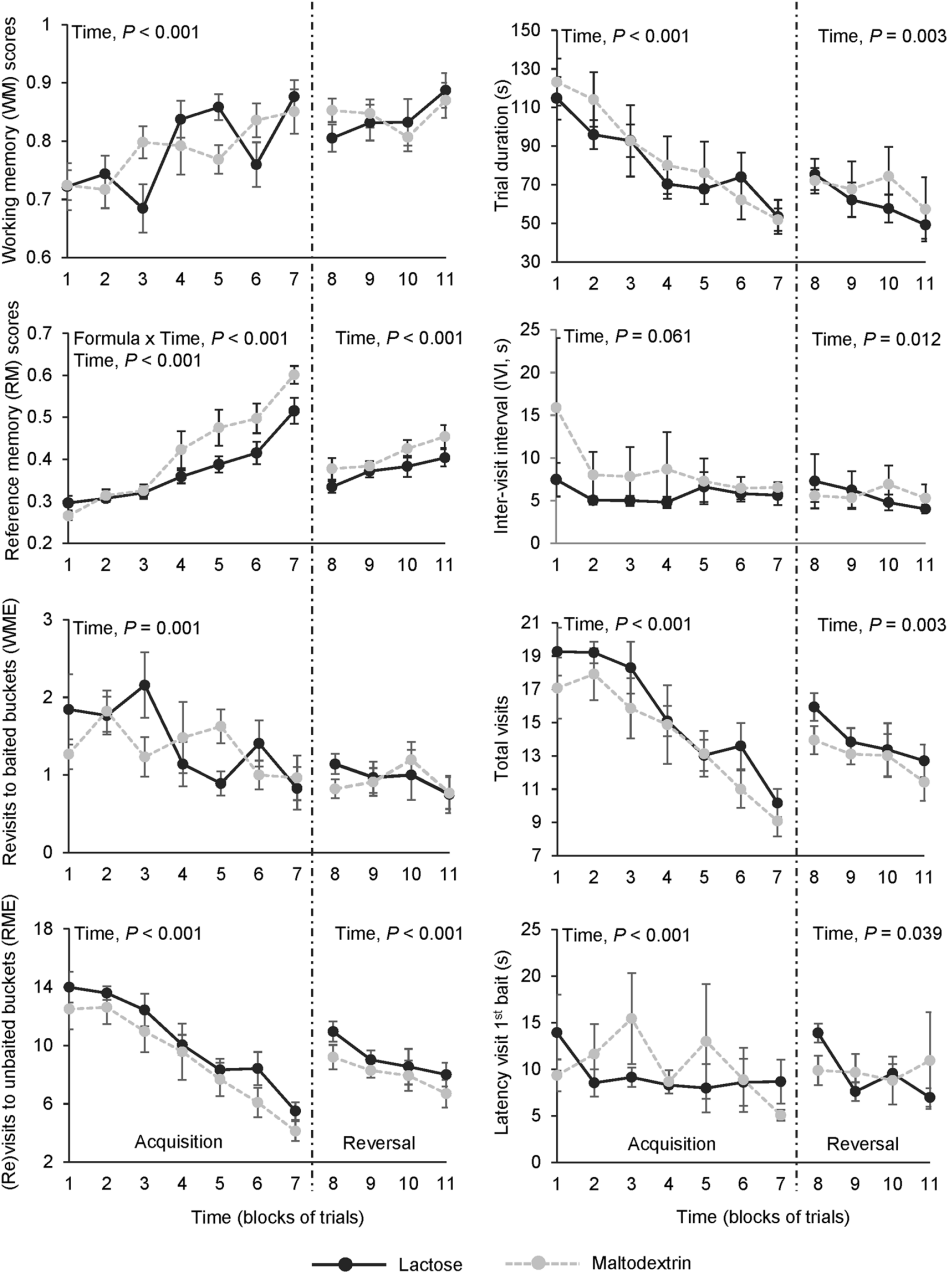
Performance of piglets fed either a lactose-based formula or a maltodextrin-based formula from 1 to 9 weeks of age, in the acquisition and reversal phases of a spatial holeboard task. Each block of trials (*i.e*. time) represents the average of 4 consecutive trials. Data are expressed as means ± SEM and were analysed with a linear mixed model.

Performance in the acquisition phase was significantly affected by time (*i.e*. blocks of trials; Fig. 3). WM and RM scores increased linearly, while WM and RM errors, trial duration and total number of visits decreased linearly over time (*p* < 0.001 for all). RM scores were significantly affected by formula × time interactions (*p* < 0.001), with piglets fed the maltodextrin-based formula having better RM scores than piglets fed the lactose-based formula towards the end of the acquisition phase. Formula had no effect on the other variables during the acquisition phase (*p* > 0.10 for all).

#### Transition phase

**Figure 4:**
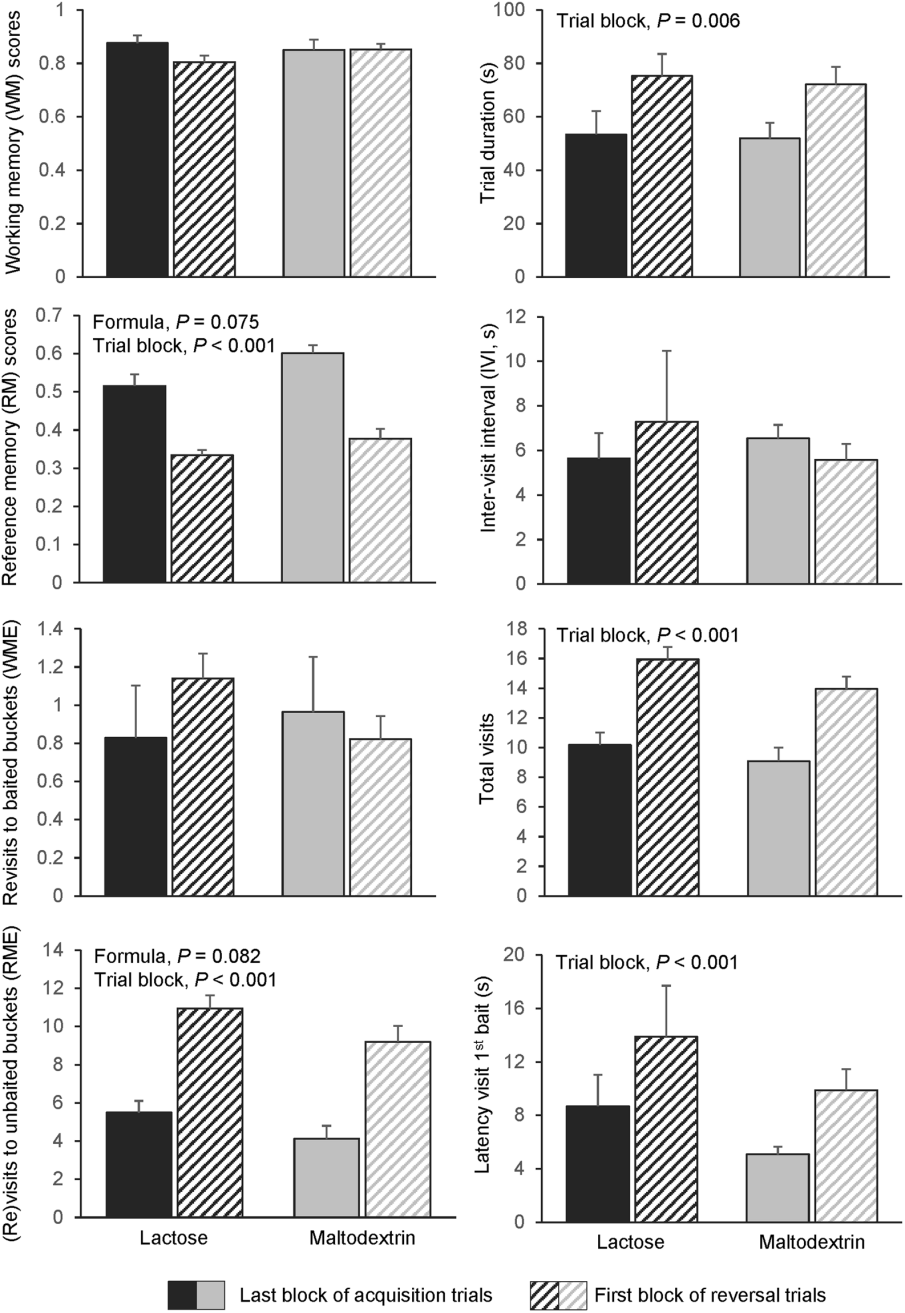
Changes of performance between the last block of 4 acquisition trials and the first block of 4 reversal trials in the spatial holeboard task, in piglets fed either a lactose-based formula or a maltodextrin-based formula from 1 to 9 weeks of age. During the reversal phase, the configuration of rewarded buckets was different than during the acquisition phase. The change of performance after the switch of configuration reflect the cognitive flexibility of the piglets. A block of trials represents the average of 4 consecutive trials. Data are expressed as means ± SEM and were analysed with a mixed model.

Piglets had lower RM scores (*p* < 0.001) and made more RM errors (*p* < 0.001) during the first block of reversal trials than during the last block of acquisition trials (transition phase; Fig. 4). WM scores and errors, however, remained unchanged (*p* > 0.10). Trial duration (*p* = 0.006) and latency to first baited visits (*p* < 0.001) were significantly longer, and more visits were made (*p* < 0.001) during the first block of reversal trials than during the last block of acquisition trials. During this transition phase, piglets fed the maltodextrin-based formula tended to have better RM scores (*p* = 0.08), and to make fewer RM errors than piglets fed the lactose-based formula (*p* = 0.08). No effects of the formula × trial block interaction were found on any of the variables.

#### Reversal phase

After the drop in RM performance after the switch of configuration, RM scores (*p* < 0.001) increased linearly, while the number of RM errors (*p* < 0.001) decreased linearly over time (*i.e*. block of trials) during the reversal phase (Fig. 3). The WM scores and WM errors were not affected by time (*p* > 0.10). Trial duration (*p* = 0.003), IVI (*p* = 0.01), total visits (*p* = 0.003), and latency to first baited visit (*p* = 0.04) decreased linearly over time during the reversal phase. Formula or its interaction with time had no effects on any of the variables during the reversal phase (*p* > 0.10 for all).

### Single-solution consumption test

**Figure 5:**
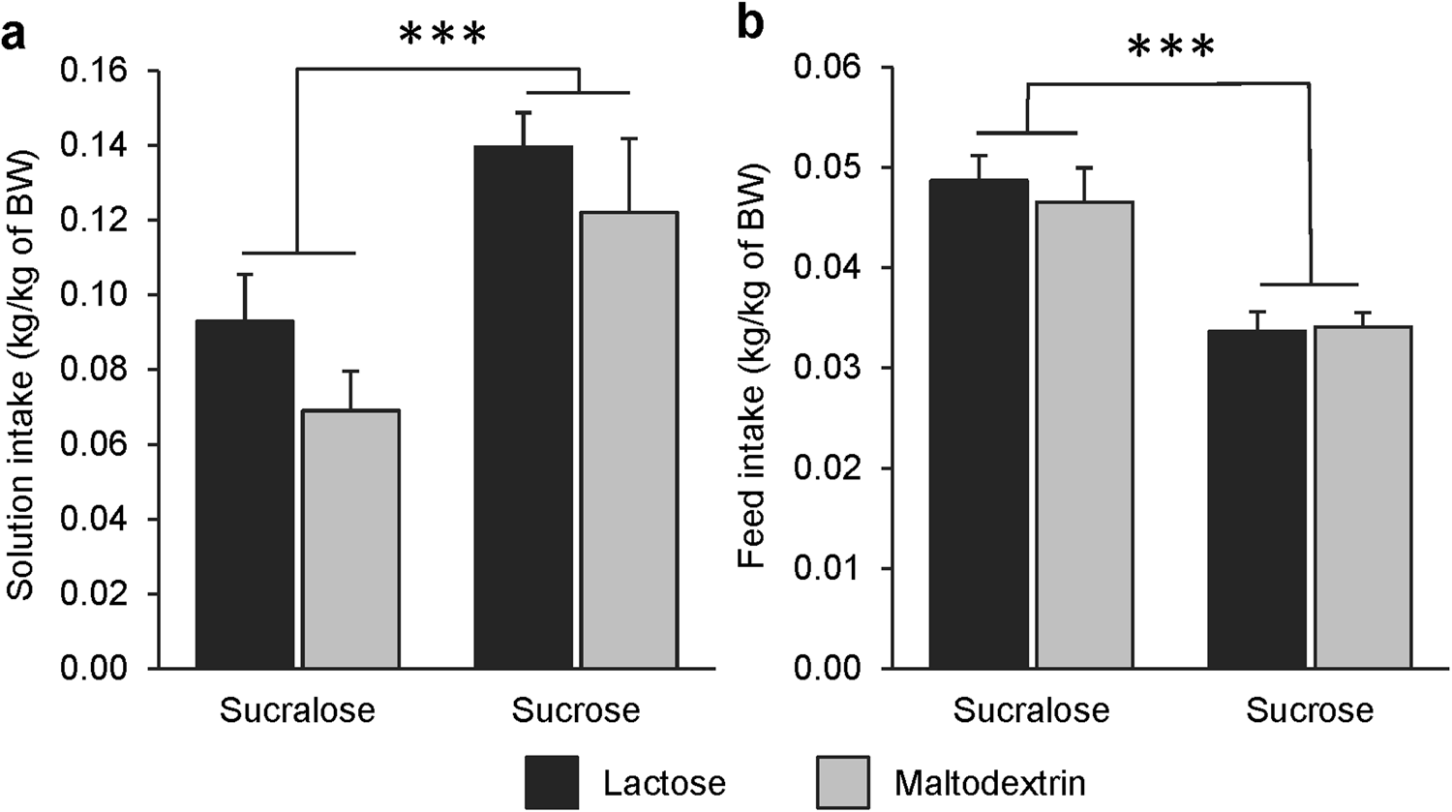
(**a**) Intake (kg/kg of body weight) of sucralose or sucrose solutions offered during 30-min single-solution tests to piglets fed either a lactose-based formula or a maltodextrin-based formula from 1 to 9 weeks of age. (**b**) Intake (kg/kg of body weight) of feed during the first 4 hours following the distribution of the solutions. Data are expressed as means ± SEM and were analysed with a mixed model. ***p < 0.001 (main effect of solution).

Irrespective of the formula, piglets drank more of the sucrose solution than of the sucralose solution during the test (*p* < 0.001; Fig. 5a). Piglets consumed significantly less feed after the test with the (caloric) sucrose solution than after the test with the (non-caloric) sucralose solution (*p* < 0.001; Fig. 5b). The reduced feed intake of pigs tested with the sucrose solution resulted in relatively similar total energy intake (from solution and feed) between pigs tested with the sucrose solution and pigs tested with the sucralose solution (135 *vs*. 116 kcal/kg of body weight). Formula and its interaction with solution had no effect on solution intake during the test (*p* > 0.10 for both), or feed intake after the test (*p* > 0.10 for both).

## Discussion

We described, for the first time, beneficial effects of using maltodextrin, compared to lactose, as the main carbohydrate source in milk formulas on later life cognitive functions of piglets, used as a model for human infants. The results of our study indicate that consuming a maltodextrin-based formula, *vs*. a lactose-based formula, from 1 to 9 weeks of age, improves reference (*i.e*. long-term) memory in a spatial holeboard task, even weeks after the end of the intervention. It is worth noting that, although maltodextrin-fed piglets were slightly lighter than lactose-fed piglets over the period following separation from the sow, the type of carbohydrates contained in the milk formulas had no impact on piglets’ growth rates over the course of the trial, which corroborates findings in infants fed maltodextrin- *vs*. lactose based milk formulas^7^.

During the acquisition phase of the holeboard task, RM and WM scores improved, while errors, latency to first reward, and trial duration decreased linearly over time, indicating that (young) piglets were able to learn the task, as previously reported^19,28–30^. Piglets fed the maltodextrin-based formula showed, towards the end of acquisition, higher RM scores than piglets fed the lactose-based formula. Piglets fed the maltodextrin-based formula also tended to show higher RM scores and made fewer RM errors than piglets fed the lactose-based formula during the transition phase, *i.e*. during the switch to a ‘reversed’ configuration of baited buckets. The levels of performance found in both groups are within the ranges of performance reported in prior research using pigs of the same age^19,28–30^.

A factor that might have affected the performance of the piglets in the holeboard task could be differences in stress levels between the two experimental groups. Stress is known to have a significant impact on spatial learning and memory^31^, and consumption of carbohydrates (and refined sugars) has been found to influence anxiety and stress in humans^32^ and animals^33,34^. In our study, however, piglets were extensively habituated to the experimenters, the apparatus, and the task – which is also a non-aversive spatial learning paradigm – before the start of the acquisition phase. After this phase of habituation, piglets selected for testing did not show any extreme stress responses, such as escape attempts, suggesting that differences in stress levels between groups were very unlikely to explain the effect of the formula on RM performance.

No effects of the formula were found on IVI and trial duration, during either the acquisition or transition phase. These results indicate that animals were equally motivated and physically able to perform the task^19,27^, and that differences of performance were not due to motivational factors or locomotor problems. The absence of motivational bias for the sweet food rewards in the holeboard is supported by data from the single-solution consumption tests conducted after the end of the holeboard test, in which the formula had no effect on the voluntary intake of sweet caloric (sucrose) or sweet non-caloric (sucralose) solutions. As early life carbohydrates play an important role in the development of food preferences and the consumption of sweet, high-energy food later in life^16^, we expected that the intake of formulas containing different carbohydrates in early life would influence preference for sweet caloric or sweet non-caloric compounds later in life. Contrarily to our postulate, however, replacing lactose by maltodextrin in milk formulas did not alter long-term preferences (up to 8 weeks after the end of the intervention) of piglets for sweet, caloric beverages; differences in motivation for the sweet, caloric food rewards used in the holeboard (chocolate raisins) were, therefore, very unlikely. Interestingly, piglets drank more of the sucrose solution than of the sucralose solution during the test, irrespective of the formulas offered in early life. This is consistent with studies showing that sucrose is the most preferred sugar for piglets, at various concentrations^35,36^. The sweet taste of sucralose can also elicit preference responses, but of weaker intensity^36^. Piglets also consumed significantly less feed after the test with the (caloric) sucrose solution than after the test with the (non-caloric) sucralose solution. The lower feed intake of pigs tested with the sucrose solution resulted in relatively similar total (solution and feed) energy intake between pigs tested with sucrose and pigs tested with sucralose solutions, thus illustrating their propensity to regulate their intake based on energy^37^.

As neither motivation, locomotion or stress seem to explain the differences of performance found in the holeboard task, it is safe to assume that the higher RM performance of the piglets fed the maltodextrin-based formula in the holeboard task reflects enhanced spatial long-term memory *per se*. The effect of the formulas on long-term memory performance could be linked to differences in glucose availability in early life. Under physiologic conditions, glucose is the primary energy source for the growth (and functioning) of the immature brain in humans and animal models^14^, and poor glucose regulation has been associated with impaired cognitive function in humans^38^. Replacing lactose by glucose syrup (DE 24), a starch derivative, in infant formulas has been found to modify metabolic post-prandial responses of formula-fed infants with, notably, slightly lower blood glucose levels 120 min after the meal^17^. It is thus possible that, in our study, persistent alterations of glucose metabolism in the early postnatal period shaped brain development and long-term cognitive function of piglets fed the maltodextrin-based formula, possibly by impacting the supply of fuel needed for brain development. Accordingly, metabolic data collected on the same animals in respiratory chambers (between 7 and 9 weeks of age) showed that piglets fed the maltodextrin-based formula had higher post-prandial rates of carbohydrate oxidation than piglets fed the lactose-based formula (article in preparation). These higher rates of carbohydrate oxidation might reflect increased utilization of glucose by the immature brain for its growth, thereby enhancing early brain development and cognitive functions later in life. Further investigation is however needed to confirm or disprove this postulate or determine whether these cognitive effects rely on other metabolic alterations induced by the milk formulas. For instance, infants fed formulas containing glucose syrup, a starch derivative, have been found to have lower post-prandial levels of circulating ketones than lactose-fed infants^17^. Ketones are a major source of energy for brain growth and metabolism, notably in hypoglycemic conditions^14^, which suggests that changes in fat metabolism may also be involved in the effect of early carbohydrate source on cognition.

Although the mechanisms underlying these effects remain unclear, carbohydrate sources in early life impacted the RM performance during acquisition, and tended to do so during the transition phase, whereas WM were unaffected. This finding is consistent with prior research showing differential effects of early life factors (*e.g*. dietary iron deficiency, low birth weight) on WM and RM performance^28,39,40^, which are two independent components of spatial memory^41^. The hippocampus is thought to be required for both reference and working memory when it comes to spatial cognition^42,43^. Lesion studies, however, indicate differential effects of hippocampal alterations on RM and WM. Inactivation of the dorsal hippocampus has been found to increase the number of RM errors made by rats in a delayed alternation task. In contrast, inactivation of the medial prefrontal cortex impaired spatial WM, but not RM performance^44^. Lesions of the hippocampus, but not of the prefrontal cortex, also impaired spatial RM performance of rats in a water maze^45,46^. That RM, but not WM, was affected by the source of carbohydrates in our study may indicate that the early nutritional intervention impacted hippocampal development, in particular. The duration of postnatal hippocampal growth in pigs is significantly longer than that of the cortex, *i.e*. approximately 9 *vs*. 6 weeks^47^, with a maximum growth rate occurring between 3 and 8 weeks of age. The critical windows for early nutritional influences is, therefore, larger for the development of the hippocampus than that of cortical structures, such as the prefrontal cortex, which may render the hippocampus more sensitive to early nutritional interventions.

Piglets took more time to finish the trial and to find the first reward, made more visits and more RM errors, and had lower RM scores during the first reversal trials (*i.e*. after the switch of configuration) than during the last acquisition trials, irrespective of the milk formula. This drop in performance illustrates the difficulty for the piglets to adapt to an unexpected change in location of the baits, as reported previously^39^. Interestingly, compared to the lactose-fed piglets, piglets fed the maltodextrin-based formula also tended to have higher RM scores and to make fewer RM errors during the transition phase (*i.e*. last acquisition block and first reversal block), and not only during acquisition. However, the better RM performance in the transition phase was likely mainly explained by the higher RM scores of the maltodextrin-fed piglets at the end of the acquisition phase, suggesting that the early life carbohydrate source had little to no effect on cognitive flexibility, that is the ability of piglets to cope with an unexpected change in the task^27,39^. Visual analysis of the reversal data, however, showed that piglets fed the maltodextrin-based formula appeared to make (numerically) fewer RME and fewer total visits, and to find the first reward faster than piglets fed the lactose-based formula in the first block of reversal trials (Fig. 2); the effect was, however, not statistically significant (statistics not shown), and replicate studies on a larger set of animals are warranted to confirm or disprove a potential effect of the milk formulas on cognitive flexibility.

In conclusion, piglets fed the maltodextrin-based formula showed improvement of spatial reference, *i.e*. long-term, memory and, to a lesser extent, cognitive flexibility in a holeboard task, even weeks after the end of the dietary intervention. Using maltodextrin, compared to lactose, as the main carbohydrate source in the milk formulas fed in early life did not affect growth or long-term preferences for sweet caloric or non-caloric beverages. This is the first study showing, in the porcine model, that maltodextrin in milk formulas can stimulate neurocognitive development compared to lactose. Further studies are warranted to unravel the mechanisms, at the central (alterations of hippocampal development) and peripheral levels (changes in post-prandial glycaemic responses), underlying the beneficial effects of maltodextrin-based formulas on cognition.
